# The Association Between Serum Isthmin-1 and Disease Activity, Inflammation, and Autoantibody Status in Rheumatoid Arthritis

**DOI:** 10.3390/diagnostics15111316

**Published:** 2025-05-23

**Authors:** Burak Oz, Ibrahım Gunduz, Gulsah Yamancan, Yusuf Dogan, Ramazan Fazıl Akkoc, Nevzat Gozel, Mustafa Gur, Ahmet Karatas, Suleyman Serdar Koca

**Affiliations:** 1 Department of Rheumatology, Faculty of Medicine, Firat University, Elazig 23119, Turkey; gulsahaydn@windowslive.com (G.Y.); yusuf.230@hotmail.com (Y.D.); mustafagur@mail.com (M.G.); drakaratas@yahoo.com (A.K.); kocassk@yahoo.com (S.S.K.); 2 Department of Rheumatology, Selahaddin Eyyubi State Hospital, Diyarbakir 21070, Turkey; abrahim724gunduz@hotmail.com; 3 Department of Anatomy, Faculty of Medicine, Firat University, Elazig 23119, Turkey; ramazan_fazil@hotmail.com; 4 Department of Internal Medicine, Faculty of Medicine, Firat University, Elazig 23119, Turkey; drngozel@hotmail.com

**Keywords:** rheumatoid arthritis, Isthmin-1, disease activity, autoantibodies, DAS28, biomarker, adipokine

## Abstract

**Background/Objectives**: Isthmin-1 (ISM1) is a secreted protein involved in immune regulation, inflammation, and angiogenesis. Although ISM1 has been implicated in chronic inflammatory conditions, its clinical relevance in rheumatoid arthritis (RA) remains unknown. This study aimed to evaluate serum ISM1 levels in RA patients and assess their associations with disease activity, autoantibody status, and inflammatory markers. **Methods**: This cross-sectional study included 90 RA patients fulfilling the 2010 ACR/EULAR criteria and 30 age- and sex-matched healthy controls. Serum ISM1 concentrations were measured using ELISA. Disease activity was assessed using DAS28-CRP and DAS28-ESR. Statistical analyses included group comparisons, correlation testing, multivariate linear regression, and ROC curve analysis to evaluate the predictive performance of ISM1 for remission or low disease activity. **Results**: Serum ISM1 levels were significantly lower in RA patients than in controls (454 ± 378 vs. 972 ± 809 ng/L, *p* < 0.001). ISM1 concentrations were inversely correlated with CRP, ESR, and both DAS28 indices. Multivariate regression confirmed independent associations between lower ISM1 concentrations and higher disease activity. ISM1 levels were significantly reduced in RF- and anti-CCP-positive patients, as well as in treatment-naïve early RA. ROC analysis identified a cut-off value of 673.73 ng/L for predicting remission or low disease activity, with an AUC of 0.713 (95% CI: 0.596–0.820), 100% specificity, and 38.9% sensitivity. **Conclusions**: This study is the first to demonstrate that serum ISM1 is independently associated with disease activity and autoantibody positivity in RA. High ISM1 levels may serve as a specific indicator of clinical remission or low disease activity, supporting its potential as a non-invasive biomarker for disease monitoring.

## 1. Introduction

Rheumatoid arthritis (RA) is a chronic autoimmune disease characterized by synovial inflammation, joint destruction, and systemic complications, significantly affecting patients’ quality of life [1]. Disease pathogenesis involves complex interactions between genetic predisposition, environmental factors, and immune system dysregulation, leading to inflammation and autoantibody production [2]. Despite advances in therapeutic strategies, achieving sustained remission remains challenging, necessitating the identification of novel biomarkers for improved management of disease activity and progression.

Isthmin-1 (ISM1) is a secreted protein originally identified in the midbrain–hindbrain boundary of Xenopus embryos, playing roles in developmental biology [3]. Recent studies indicate ISM1 as an adipokine involved in metabolic processes, inflammatory regulation, and immune response modulation [4,5]. ISM1 is expressed across various tissues, including adipocytes, skin, mucosal surfaces, and immune cells such as NK, NKT, and Th17 cells, suggesting its potential multifaceted biological roles [6,7].

Emerging evidence highlights the association between ISM1 and several pathophysiological conditions, including glucose metabolism disorders, immune dysregulation, inflammation, and cancer progression [5,7]. ISM1 regulates glucose uptake via the translocation of glucose transporter GLUT4, enhancing glycolysis, and influencing inflammatory responses through the modulation of Nuclear Factor Kappa B (NF-κB) and inflammatory cytokines [4,8]. Furthermore, recent findings suggest ISM1’s role in aging-related cardiac dysfunction, exerting protective effects by enhancing glycolysis and activating SIRT1 deacetylase activity [4].

ISM1 exerts anti-inflammatory, immunomodulatory, and anti-angiogenic effects through multiple mechanisms, including the suppression of NF-κB signaling, modulation of Th17 cell responses, enhancement of efferocytosis, and inhibition of endothelial proliferation [4,5,6,8,9]. These mechanisms are relevant to the pathogenesis of RA, which is characterized by synovial inflammation, immune dysregulation, and aberrant angiogenesis [1,10,11]. In view of this biological alignment, ISM1 may be considered a pathophysiological relevant molecule in RA. To our knowledge, serum ISM1 levels have not yet been clinically evaluated in RA, and their relationship with systemic inflammation, disease activity, or autoantibody status remains unexplored. Consequently, the present study hypothesized that serum ISM1 levels would be inversely associated with disease activity and immune activation in RA, potentially serving as a novel biomarker for disease monitoring.

## 2. Materials and Methods

This cross-sectional study aimed to evaluate serum ISM1 levels and their relationship with disease activity in RA patients. The study protocol was reviewed and approved by the University Non-Interventional Research Ethics Committee (approval number: 20.05.2024/24299). Written informed consent was obtained from all participants prior to enrolment.

A total of 120 participants were enrolled, including 90 patients diagnosed with RA according to the 2010 ACR/EULAR classification criteria and 30 age- and sex-matched healthy controls. RA patients were further stratified into moderate-to-high (DAS28-CRP ≥ 3.2, *n* = 54; DAS28-ESR ≥ 3.2, *n* = 59) and remission-to-low disease activity groups (DAS28-CRP < 3.2, *n* = 36; DAS28-ESR < 3.2, *n* = 31). Patients were also categorized according to disease duration as early RA (≤12 weeks; *n* = 30) and established RA (>12 weeks; *n* = 60).

Fasting blood samples were collected to measure biochemical parameters including glucose, aspartate aminotransferase (AST), alanine aminotransferase (ALT), urea, creatinine, uric acid, total protein, albumin, hemoglobin (Hb), white blood cells (WBCs), lymphocytes (Lym), platelets, C-reactive protein (CRP), erythrocyte sedimentation rate (ESR), rheumatoid factor (RF), and anti-cyclic citrullinated peptide (anti-CCP) antibodies. Serum ISM1 concentrations were measured using a commercially available Human ISM1 ELISA Kit (catalogue no.: 201-12-4050; Sunred Biological Technology Co., Ltd., Shanghai, China), following the manufacturer’s instructions. The assay range was 10–3000 ng/L, with a minimum detectable concentration (sensitivity) of 9.625 ng/L. The intra-assay coefficient of variation (CV) was <10%, and the inter-assay CV was <12%.

Descriptive statistics are presented as the mean ± standard deviation (SD), median, IQR (interquartile range: 25th–75th percentiles), and 95% confidence interval (CI). Normality of data distribution was assessed using the Kolmogorov–Smirnov test. Between-group comparisons were performed using an independent samples *t*-test, chi-square test, or Mann–Whitney U test, as appropriate. Comparisons involving more than two groups were analyzed using the Kruskal–Wallis test, followed by post hoc pairwise analyses with the Dwass–Steel–Critchlow–Fligner method. Correlations between serum ISM1 levels and clinical parameters were assessed using Spearman’s rank correlation analysis. Effect sizes were calculated using Cohen’s d for group comparisons. Effect sizes (Cohen’s d) were interpreted according to conventional thresholds, small (≥0.20), moderate (≥0.50), large (≥0.80), and very large (≥1.30), to provide additional context regarding the magnitude of group differences. Statistical significance was defined as *p*-values less than 0.05. Data analyses were performed using Jamovi version 2.3.28 (The Jamovi project, 2024). Post hoc power analysis was performed using G*Power version 3.1.9.2 (Franz Faul, Universität Kiel, Kiel, Germany), a freely available statistical software for power calculations. The analysis targeted the primary comparison of serum ISM1 levels between RA patients and healthy controls. Based on the observed effect size (Cohen’s d = 1.00), group sample sizes (*n* = 90 for RA; *n* = 30 for controls), and a two-tailed significance level (α = 0.05), the resulting statistical power was 0.997 (99.7%), indicating a highly adequate sample size for detecting the observed group difference.

## 3. Results

### 3.1. Demographic and Clinical Characteristics

Demographic, clinical, and laboratory parameters were compared between RA patients and healthy control subjects (Table 1). There were no significant differences in age, gender distribution, body mass index (BMI), and routine biochemical parameters such as glucose, AST, ALT, urea, creatinine, uric acid, total protein, albumin, WBCs, lymphocyte count, and platelet count between RA patients and controls (*p* > 0.05 for all comparisons). However, Hb levels were significantly lower (12.8 ± 1.51 (12.8) vs. 13.5 ± 0.38 (13.6) g/dL, *p* = 0.001), while inflammatory markers, CRP (13.7 ± 20.3 (7.18) vs. 4.57 ± 1.30 (4.0) mg/L, *p* = 0.004) and ESR (30.1 ± 18.4 (29) vs. 16.7 ± 3.55 (16) mm/h, *p* < 0.001), were significantly higher in the RA group compared with controls. Serum ISM1 levels were significantly lower in RA patients compared to controls (454 ± 378 (342) vs. 972 ± 809 (678) ng/L, *p* < 0.001).

### 3.2. ISM1 Levels and Autoantibody Status

RF-positive patients had significantly reduced ISM1 levels (mean ± SD, 383 ± 286 ng/L; median (IQR), 312 (160) ng/L) compared to RF-negative patients (mean ± SD, 589 ± 487 ng/L; median (IQR), 421 (384) ng/L; *p* = 0.004), with a moderate effect size (Cohen’s d = 0.56).

Similarly, anti-CCP-positive patients showed significantly lower ISM1 concentrations (mean ± SD, 385 ± 247 ng/L; median (IQR), 329 (171) ng/L) compared to anti-CCP-negative individuals (mean ± SD, 750 ± 637 ng/L; median (IQR), 437 (697) ng/L; *p* = 0.007), accompanied by a large effect size (Cohen’s d = 1.09).

Furthermore, patients who were double-positive for RF and anti-CCP antibodies also exhibited significantly lower serum ISM1 levels (mean ± SD, 333 ± 176 ng/L; median (IQR), 303 (150) ng/L) compared to double-negative patients (mean ± SD, 653 ± 518 ng/L; median (IQR), 443 (539) ng/L; *p* < 0.001), with a large effect size (Cohen’s d = 0.95) (Table 2). As summarized in Table 2, the observed effect sizes ranged from moderate (RF-positive vs. negative) to large (anti-CCP and double-positive vs. double-negative), underscoring the clinical relevance of ISM1 reductions in autoantibody-positive RA.

### 3.3. ISM1 Levels According to Disease Activity

**Figure 1:**
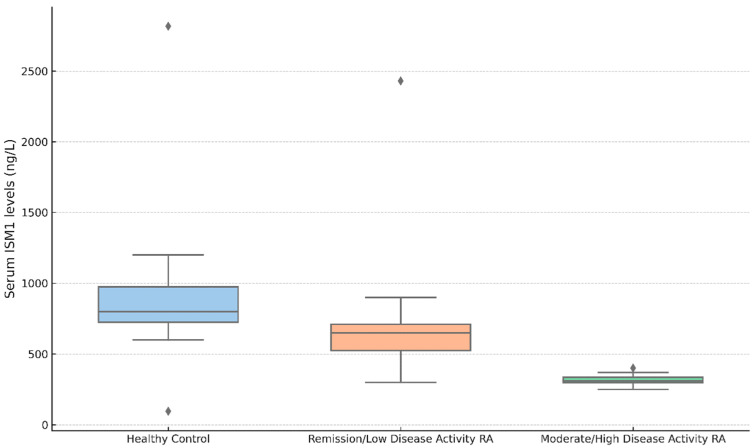
Serum ISM1 levels in healthy controls and RA patients stratified by disease activity (DAS28-CRP); boxplot showing serum ISM1 levels (ng/L) in healthy controls (*n* = 30) and RA patients stratified by DAS28-CRP scores: remission/low (*n* = 36) and moderate/high activity (*n* = 54). The Kruskal–Wallis test revealed significant differences (χ^2^ = 34.0, *p* < 0.001; ε^2^ = 0.285). Post hoc analysis showed lower ISM1 levels in the moderate/high group compared to both controls (*p* < 0.001) and the remission/low group (*p* = 0.002); the difference between the controls and the remission/low group was not significant (*p* = 0.104).

Serum ISM1 levels differed significantly among RA patients stratified by disease activity and healthy controls (Table 3, Figure 1). Healthy individuals exhibited markedly higher ISM1 concentrations (mean ± SD, 972 ± 809 ng/L; median (IQR), 678 (710) ng/L) compared to RA patients with moderate-to-high disease activity defined by DAS28-CRP ≥ 3.2 (mean ± SD, 315 ± 125 ng/L; median (IQR), 311 (184) ng/L; *p* < 0.001), with a very large effect size (Cohen’s d = 1.34). A significant difference was also observed between the high activity group and patients in remission or with low disease activity (DAS28-CRP < 3.2: 663 ± 515 ng/L; median (IQR), 407 (584) ng/L; *p* = 0.002), with a large effect size (Cohen’s d = 1.03). Although ISM1 levels appeared to be reduced in the low activity group compared with healthy controls, this difference did not reach statistical significance (*p* = 0.104; Cohen’s d = 0.47).

Similarly, when disease activity was assessed using DAS28-ESR, patients with moderate-to-high activity (≥3.2) had significantly lower serum ISM1 levels (mean ± SD, 317 ± 116 ng/L; median (IQR), 306 (165) ng/L) compared to those in remission or with low disease activity (<3.2: 716 ± 539 ng/L; median (IQR), 475 (654) ng/L; *p* < 0.001), with a large effect size (Cohen’s d = 1.22). The high activity group also showed significantly lower ISM1 levels compared to the healthy controls (*p* < 0.001; Cohen’s d = 1.37), whereas the difference between the low activity group and the controls was not statistically significant (*p* = 0.334; Cohen’s d = 0.37).

These differences were confirmed using the Kruskal–Wallis test (χ^2^ = 34.0, *p* < 0.001; ε^2^ = 0.285), with post hoc pairwise comparisons conducted via the Dwass–Steel–Critchlow–Fligner method. Collectively, these findings indicate a strong inverse relationship between serum ISM1 levels and RA disease activity, particularly when assessed using inflammatory indices such as CRP and ESR, and demonstrate that the observed differences are not only statistically significant but also clinically meaningful. As summarized in Table 3, effect sizes were large to very large across disease activity strata, further supporting the clinical impact of ISM1 suppression in active RA.

### 3.4. ISM1 Levels According to Disease Duration

**Figure 2:**
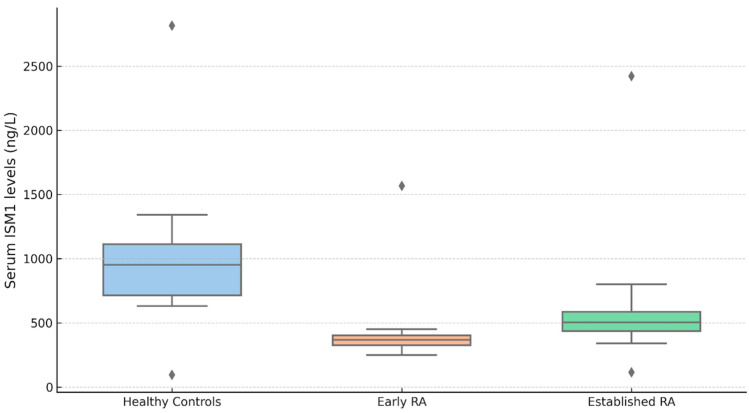
Serum ISM1 levels in healthy controls and RA patients by disease duration; Serum ISM1 levels (ng/L) in healthy controls (*n* = 30), early RA (≤12 weeks; *n* = 30), and established RA (>12 weeks; *n* = 60). The Kruskal–Wallis test showed significant group differences (χ^2^ (2) = 21.9, *p* < 0.001; ε^2^ = 0.184). The post hoc Dwass–Steel–Critchlow–Fligner test revealed lower ISM1 levels in both RA groups vs. controls (*p* < 0.001), with no difference between RA subgroups (*p* = 0.962). Data are shown as the median (IQR); outliers are displayed as individual points. Serum ISM1 levels–median (IQR): healthy controls: 678 (710), early RA (≤12 weeks): 347 (142), established RA (>12 weeks): 341 (256).

Serum ISM1 levels also varied significantly according to disease duration in RA patients compared with healthy controls (Figure 2). The Kruskal–Wallis test indicated a significant overall difference among the three groups (χ^2^(2) = 21.9, *p* < 0.001; ε^2^ = 0.184). Healthy controls exhibited markedly higher serum ISM1 levels (median (IQR), 678 (710) ng/L) than both early RA patients (≤12 weeks) (347 (142) ng/L; *p* < 0.001) and established RA patients (>12 weeks) (341 (256) ng/L; *p* < 0.001). However, no statistically significant difference in ISM1 concentrations was observed between the early and established RA groups (*p* = 0.962). These results suggest that the reduction in serum ISM1 levels occurs early in the disease course and remains stable over time, irrespective of the duration of the disease. Importantly, patients in the early RA group (≤12 weeks) included in our study were treatment-naïve, having not received any cDMARDs or biologic agents at the time of serum sampling. This strengthens the interpretation that the observed reduction in ISM1 levels in this group may reflect the natural pathophysiological alterations associated with untreated early disease, rather than being confounded by therapeutic interventions. Therefore, ISM1 suppression appears to occur early in the disease course, independent of pharmacologic modulation.

### 3.5. Correlation Between Serum ISM1 Levels and Inflammatory and Disease Activity Markers

Spearman’s rank correlation analysis demonstrated significant inverse associations between serum ISM1 levels and markers of systemic inflammation and disease activity in RA patients (Table 4). ISM1 concentrations showed a moderate negative correlation with ESR (r_s_ = −0.291, *p* = 0.001) and CRP (r_s_ = −0.342, *p* < 0.001). Furthermore, serum ISM1 levels were negatively correlated with disease activity scores, including DAS28-ESR (r_s_ = −0.385, *p* < 0.001) and DAS28-CRP (r_s_ = −0.405, *p* < 0.001). These findings indicate that lower ISM1 levels are associated with higher levels of systemic inflammation and increased clinical disease activity in RA.

To further investigate the independent association between serum ISM1 levels and disease activity, we conducted two separate multivariate linear regression models incorporating demographic (age, gender, BMI) and inflammatory covariates (hemoglobin, CRP, and ESR) (Table 5). In the first model, DAS28-CRP emerged as a significant independent predictor of lower serum ISM1 levels (β = −147.76, 95% CI: −217.80 to −77.72, *p* < 0.001), while the other covariates did not reach statistical significance. In the second model, in which DAS28-ESR was used instead of DAS28-CRP, DAS28-ESR remained a strong inverse predictor (β = −151.32, 95% CI: −216.52 to −86.13, *p* < 0.001), with other covariates again remaining non-significant.

### 3.6. Predictive Value of Serum ISM1 Levels for Low Disease Activity

To determine the potential clinical utility of ISM1 as a biomarker for disease activity stratification, a receiver operating characteristic (ROC) analysis was performed. The area under the ROC curve (AUC) for distinguishing patients in remission or with low disease activity was 0.713, with a 95% CI of 0.596–0.820, indicating moderate discriminatory performance.

**Figure 3:**
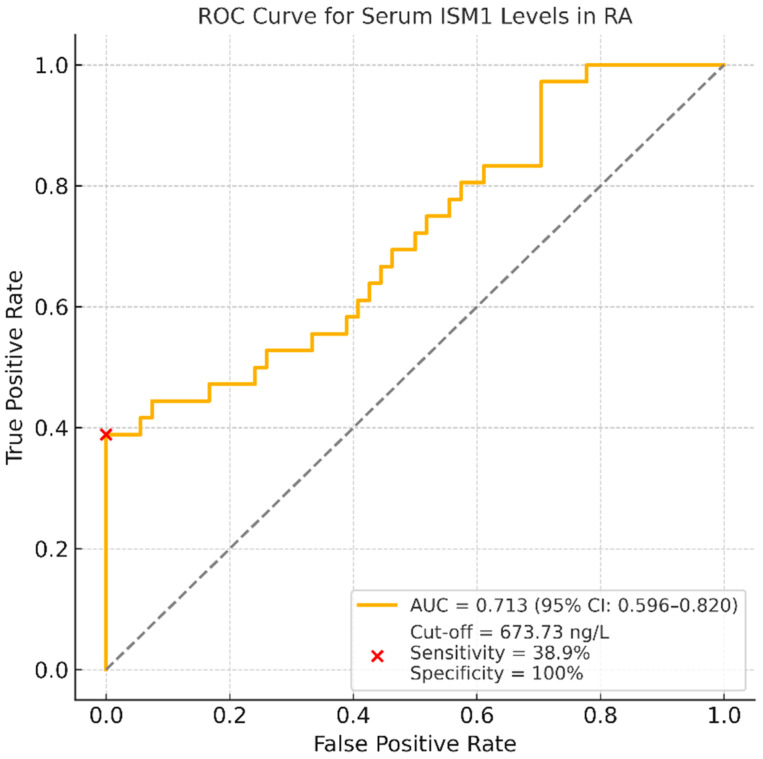
ROC curve for serum ISM1 levels distinguishing RA patients in remission or with low disease activity from those with moderate-to-high disease activity; the AUC was 0.713 (95% CI: 0.596–0.820). At the optimal cut-off value of 673.73 ng/L, serum ISM1 levels showed a sensitivity of 38.9% and a specificity of 100.0%. These findings suggest that higher ISM1 levels may help identify patients with controlled disease activity, albeit with limited sensitivity.

At the optimal cut-off value of 673.73 ng/L, serum ISM1 levels demonstrated a sensitivity of 38.9% (95% CI: 23.1–56.5) and a specificity of 100.0% (95% CI: 93.4–100.0). The corresponding positive predictive value was 100.0% (95% CI: 76.8–100.0), and the negative predictive value was 71.1% (95% CI: 59.5–80.9). These results suggest that high serum ISM1 levels are highly specific for identifying patients with low disease activity or in remission, albeit with limited sensitivity. The ROC curve corresponding to this analysis is presented in Figure 3.

## 4. Discussion

In this cross-sectional study, we provide the first clinical evidence that serum ISM1 levels are significantly reduced in RA patients compared to healthy controls. Moreover, ISM1 levels were inversely associated with DAS28-CRP and DAS28-ESR scores, CRP, ESR, and autoantibody positivity. These associations remained significant after adjustment for demographic and hematologic variables in multivariate regression models, suggesting an independent relationship between ISM1 and disease activity. Notably, ISM1 suppression was also evident in treatment-naïve patients with early RA, indicating that its downregulation occurs early in the disease course, possibly preceding pharmacologic intervention.

ISM1 is increasingly recognized as a secreted protein with multifaceted roles in immune modulation and inflammation. It has been shown to suppress NF-κB signaling and reduce pro-inflammatory cytokine production in models of acute and chronic lung injury, as well as modulate tissue repair and fibrosis [9,12]. In RA, aberrant activation of the NF-κB pathway contributes to synovial inflammation, immune cell recruitment, and cytokine amplification, particularly in Th17-mediated responses [10]. Therefore, the observed reduction in ISM1 levels in RA patients, particularly those with active disease, may reflect a loss of endogenous anti-inflammatory counter-regulation. Additionally, ISM1 has been implicated in endothelial function, oxidative stress, and glucose metabolism—processes that are also disrupted in RA pathophysiology [13,14,15]. Taken together, these mechanistic insights support a potential role for ISM1 not only as a biomarker of systemic inflammation but also as a contributor to RA pathogenesis.

Previous studies have characterized ISM1 as a novel adipokine, widely expressed in adipose, immune, and epithelial tissues, with roles in metabolism, inflammation, and angiogenesis [5,16]. It enhances glucose uptake, regulates lipid metabolism, and suppresses inflammatory responses by modulating NF-κB activation [9,17]. Consistent with these functions, in our study, we found that ISM1 levels negatively correlated with CRP and ESR, and positively correlated with hemoglobin. These findings support ISM1’s role as a modulator of immune and inflammatory responses in RA [6,9].

The inverse association between ISM1 and disease activity was particularly evident in patients with moderate-to-high disease activity. Moreover, our ROC analysis demonstrated that serum ISM1 levels ≥ 673.73 ng/L predicted remission or low disease activity with excellent specificity (100%) and positive predictive value (100%), although sensitivity was modest (38.9%). These findings further support the notion that elevated ISM1 levels may serve as a reliable indicator of controlled disease states in RA.

Despite the fact that serum ISM1 levels exhibited high specificity (100%) in identifying RA patients with remission or low disease activity, the sensitivity was comparatively low (38.9%) at the proposed cut-off value. This finding suggests that ISM1 may be more appropriate as a complementary biomarker rather than a primary screening tool in clinical practice. Its high specificity could support decision-making in selected clinical contexts, such as confirming remission or evaluating treatment response in conjunction with established indices like DAS28.

Furthermore, ISM1 levels were markedly lower in patients who were RF- and anti-CCP-positive, indicating a potential link between ISM1 and autoantibody-mediated immune mechanisms. Experimental studies suggest that ISM1 may influence immune tolerance by inducing apoptosis in proinflammatory macrophages, enhancing adiponectin release, and suppressing NF-κB signaling [9,18,19]. These pathways are integral to autoimmunity and chronic inflammation, reinforcing the relevance of ISM1 suppression in autoantibody-positive RA.

While the findings establish robust inverse correlations between serum ISM1 levels and inflammatory markers such as CRP, ESR, and DAS28 indices, the directionality of this relationship remains uncertain. The role of ISM1 downregulation in driving inflammation remains to be elucidated; it is unclear whether it plays an active role or merely reflects a secondary consequence of heightened immune activation in RA. Preclinical studies have demonstrated that inflammatory stimuli, including LPS exposure, can suppress ISM1 expression [9], thus suggesting that systemic inflammation may result in a reduction in ISM1 levels. In contrast, ISM1 has also been reported to attenuate inflammatory signaling via the inhibition of NF-κB, enhancement of efferocytosis, and modulation of proinflammatory cytokines [9,19]. Consequently, ISM1 suppression in RA may serve as both an indicator and a mediator of the inflammatory burden.

The fact that ISM1 levels did not differ between early and established RA, but were significantly reduced in active disease, suggests that ISM1 reflects disease burden rather than the duration of the disease. This is further supported by the presence of low ISM1 levels in treatment-naïve patients, indicating that the observed suppression is not merely a consequence of immunosuppressive therapy. These findings point to the potential utility of ISM1 as a dynamic, non-invasive biomarker for monitoring RA activity.

Beyond inflammation, ISM1 has also been implicated in angiogenesis, a key pathological feature in RA. Angiogenesis contributes to pannus formation and persistent synovial inflammation. Interestingly, ISM1 exhibits anti-angiogenic properties by inducing endothelial cell apoptosis and inhibiting neovascularization [20,21]. It is overexpressed in glomerulopathy models and impairs podocyte viability, suggesting its broader role in vascular integrity [11,13]. Although angiogenesis is essential for pannus expansion in RA [11], the role of ISM1’s anti-angiogenic activity within the inflammatory joint microenvironment remains unclear. Further studies are warranted to determine whether the suppression of ISM1 contributes to pathological angiogenesis in RA or represents a compensatory response [22].

Given the large effect sizes observed in our comparisons, ISM1 alterations appear to be clinically meaningful. The inclusion of effect size metrics such as Cohen’s d in our analysis further emphasizes the clinical relevance of ISM1 alterations in RA, beyond mere statistical significance. In light of its anti-inflammatory functions and ability to modulate NF-κB pathways, ISM1 may hold therapeutic potential in RA. Current research on NF-κB-targeted therapies underscores the importance of endogenous regulators such as ISM1 [23,24,25,26]. Whether ISM1 can be leveraged as a therapeutic agent or serve as a companion biomarker for targeted treatment strategies remains a promising area for future research.

Nevertheless, although the study was cross-sectional in design, the post hoc power analysis confirmed adequate statistical power (99.7%) for the primary outcome, supporting the reliability of our findings regarding ISM1 levels in RA.

This study has several limitations that should be acknowledged. First, due to its cross-sectional design, causal inferences regarding the relationship between ISM1 and RA disease activity cannot be drawn. Second, although treatment-naïve patients were included in the early RA group, we did not stratify established RA patients based on their current or prior medication exposure, which may potentially influence serum ISM1 levels. Third, multivariate analyses did not include treatment status, RF/anti-CCP titers, or other potentially relevant immunological parameters due to missing data and limited sample size. Additionally, serum ISM1 measurements were not complemented with synovial fluid levels or tissue expression analyses, which could have provided further mechanistic insights. Future longitudinal and multicenter studies with larger and demographically diverse RA cohorts, incorporating serial ISM1 measurements and comprehensive immunophenotyping, will be essential to validate our findings and to clarify the clinical relevance of ISM1 in RA.

## 5. Conclusions

In conclusion, this is the first clinical study to demonstrate that serum ISM1 levels are significantly reduced in RA patients and independently associated with disease activity, as measured using DAS28-CRP and DAS28-ESR, after adjustment for key inflammatory and demographic variables. Additionally, ISM1 levels were inversely associated with autoantibody positivity (RF and anti-CCP), although these relationships require confirmation in multivariate settings. ISM1 suppression was evident even in treatment-naïve early RA patients, suggesting that its downregulation occurs early in the disease course.

Importantly, ROC analysis revealed that serum ISM1 levels ≥ 673.73 ng/L could predict remission or low disease activity with excellent specificity (100%) and positive predictive value (100%). Although sensitivity was limited, this threshold may support the potential role of ISM1 as a clinically meaningful complementary indicator of controlled disease. Supported by strong effect sizes, predictive performance, and multivariate modelling, these findings highlight ISM1’s potential as a non-invasive biomarker for disease monitoring in RA. Given its established anti-inflammatory and immunomodulatory functions, ISM1 may also represent a novel target for future therapeutic research.

## Figures and Tables

**Table 1:** Comparison of demographic, clinical, and laboratory parameters between control and RA groups.

	Control Group (*n* = 30)	RA Group (*n* = 90)	*p*
Age (Years)	52.5 ± 6.43 (53.5)	54.4 ± 11.2 (56)	0.207 ^+^
Gender (Female %)	70% (*n* = 21)	72.2% (*n* = 65)	0.815 ^#^
BMI (kg/m^2^)	23.8 ± 1.18 (24)	23.9 ± 2.65 (24)	0.797 ^+^
Glucose (mg/dL)	87.3 ± 3.25 (87)	98.6 ± 36.9 (86)	0.730 ^+^
AST (U/L)	27.1 ± 7.82	24.7 ± 23.0	0.579 *
ALT (U/L)	20.8 ± 2.64 (21)	20.1 ± 5.16 (20)	0.655 ^+^
Urea (mg/dL)	28.3 ± 3.74 (29)	28.8 ± 8.68 (28)	0.745 ^+^
Creatinine (mg/dL)	0.707 ± 0.136	0.757 ± 0.282	0.348 *
Uric acid (mg/dL)	4.56 ± 0.741 (4.70)	4.50 ± 1.34 (4.35)	0.271 ^+^
Total protein (g/dL)	6.88 ± 0.225 (6.90)	6.94 ± 0.517 (7.00)	0.391 ^+^
Albumin (g/dL)	4.20 ± 0.244	4.30 ± 0.334	0.134 *
WBCs (10 × 10^3^/µL)	8613 ± 1478 (8725)	8204 ± 2782 (7720)	0.098 ^+^
Lym (10 × 10^3^/µL)	2294 ± 347 (2275)	2316 ± 525 (2250)	0.995 ^+^
Platelet (/µL)	269,833 ± 70,472	287,900 ± 98,488	0.356 *
Hemoglobin (g/dL)	13.5 ± 0.38 (13.6)	12.8 ± 1.51 (12.8)	0.001 ^+^
CRP (mg/L)	4.57 ± 1.30 (4.0)	13.7 ± 20.3 (7.18)	0.004 ^+^
ESR (mm/h)	16.7 ± 3.55 (16)	30.1 ± 18.4 (29)	<0.001 ^+^
ISM-1 (ng/L)	972 ± 809 (678)	454 ± 378 (342)	<0.001 ^+^

Mean ± SD reported for all variables; median included for non-normal data. Comparisons between groups were performed using the independent samples *t*-test (*), chi-square test (#), or Mann–Whitney U test (+), as appropriate. Abbreviations: RA, rheumatoid arthritis; BMI, body mass index; AST, aspartate transaminase; ALT, alanine transaminase; WBCs, white blood cells; Lym, lymphocytes; CRP, C-reactive protein; ESR, erythrocyte sedimentation rate; ISM1, Isthmin 1.

**Table 2:** Serum ISM1 levels according to autoantibody status in RA patients.

		ISM-1 (ng/L)			*p*	Cohen’s d
		Mean ± SD	Median	IQR		
Rf status	*Positive* (*n* = 59)	383 ± 286	312	160	0.004	0.561
	*Negative* (*n* = 31)	589 ± 487	421	384		
Anti-CCP status	*Positive* (*n* = 76)	385 ± 247	329	171	0.007	1.091
	*Negative* (*n* = 14)	750 ± 637	437	697		
Rf and Anti-CCP status	*Positive* (*n* = 58)	333 ± 176	303	150	<0.001	0.945
	*Negative* (*n* = 32)	653 ± 518	443	539		

ISM1 levels (ng/L) are presented as the mean ± SD, median, IQR (interquartile range: 25th–75th percentiles), according to RF and anti-CCP antibody status. Comparisons were performed using the Mann–Whitney U test. Effect sizes (Cohen’s d) are provided with interpretation (small ≥ 0.2, moderate ≥ 0.5, large ≥ 0.8) to indicate the magnitude of group differences. Abbreviations: Rf; rheumatoid factor, CCP; cyclic citrullinated peptide.

**Table 3:** Serum ISM1 levels in control subjects and rheumatoid arthritis patients stratified by disease activity.

					95% CI			
		Mean ± SD	IQR	Median	Lower	Upper	*p*	Cohen’s d
Healthy Control Group (*n* = 30)	*ISM1* (ng/L)	972 ± 809	710	678	670	1274		
RA (DAS28 CRP ≥ 3.2) (*n* = 54)		315 ± 125	184	311	281	349	<0.001 * 0.002 ^#^	1.337 * 1.027 ^#^
RA (DAS28 CRP < 3.2) (*n* = 36)		663 ± 515	584	407	488	837	0.104 *	
RA (DAS28 ESR ≥ 3.2) (*n* = 59)		317 ± 116	165	306	286	347	<0.001 * <0.001 ^+^	1.374 * 1.215 ^+^
RA (DAS28 ESR < 3.2) (*n* = 31)		716 ± 539	654	475	518	913	0.334 *	

Serum ISM1 levels (ng/L) are summarized as the mean ± SD, IQR (interquartile range: 25th–75th percentiles), median, and 95% CI for control subjects and RA patients grouped by DAS28-CRP scores. Groups were compared using the Kruskal–Wallis test (ε^2^ = 0.285, 0.325), with post hoc pairwise comparisons via the Dwass–Steel–Critchlow–Fligner method. Effect sizes (Cohen’s d) are provided with interpretation (small ≥ 0.2, moderate ≥ 0.5, large ≥ 0.8, and very large ≥ 1.3) to indicate the magnitude of group differences. Abbreviations: RA; rheumatoid arthritis, DAS; disease activity score, CRP; C-reactive protein; ESR, erythrocyte sedimentation rate. * vs. control, # vs. RA (DAS28-CRP < 3.2), and + vs. RA (DAS28-ESR < 3.2).

**Table 4:** Spearman correlation between serum ISM1 levels and inflammatory and hematological markers.

Variable	Spearman’s Rho (r_s_)	*p*
ESR	−0.291	0.001
CRP	−0.342	<0.001
DAS28 ESR	−0.385	<0.001
DAS28 CRP	−0.405	<0.001

Spearman’s rank correlation was used to evaluate the association between serum ISM1 levels (ng/L) and ESR, CRP, DAS28 ESR, and DAS28 CRP. Abbreviations: ESR; erythrocyte sedimentation rate, CRP; C-reactive protein, DAS; disease activity score.

**Table 5:** Multivariate linear regression analysis of serum ISM1 levels in RA patients.

Model	Predictor	β Coefficient	95% CI (Lower–Upper)	*p*	VIF
Model 1 (DAS28-CRP)	Const	1724.38	722.63 to 2726.12	0.001	195.07
	DAS28_CRP	−147.76	−217.80 to −77.72	<0.001	1.36
	Age	−3.00	−10.00 to 4.01	0.397	1.18
	Gender	8.95	−174.21 to 192.11	0.923	1.31
	BMI	−7.25	−36.15 to 21.65	0.619	1.13
	Hb	−29.05	−85.29 to 27.19	0.307	1.39
	CRP	3.50	−0.94 to 7.94	0.121	1.57
	ESR	−3.37	−8.32 to 1.58	0.179	1.60
Model 2 (DAS28-ESR)	Const	1815.66	827.43 to 2803.89	0.000	196.91
	DAS28_ESR	−151.32	−216.52 to −86.13	<0.001	1.58
	Age	−2.68	−9.54 to 4.18	0.439	1.18
	Gender	12.23	−167.59 to 192.05	0.893	1.31
	BMI	−6.44	−34.83 to 21.95	0.653	1.13
	Hb	−38.61	−93.59 to 16.37	0.166	1.38
	CRP	1.45	−2.75 to 5.66	0.494	1.46
	ESR	0.13	−5.29 to 5.56	0.962	2.00

Multivariate linear regression models were constructed using either DAS28-CRP or DAS28-ESR as the primary predictor alongside age, gender, BMI, hemoglobin, CRP, and ESR. All VIF values < 2, indicating no multicollinearity.

## Data Availability

The data supporting the findings of this study are not publicly available due to ethical and privacy restrictions but may be provided by the corresponding author upon reasonable request.

## Abbreviations

The following abbreviations are used in this manuscript:

AUC	Area Under the Curve
BMI	Body Mass Index
CI	Confidence Interval
CRP	C-Reactive Protein
DAS28	Disease Activity Score in 28 Joints
DMARDs	Disease-Modifying Anti-Rheumatic Drugs
ELISA	Enzyme-Linked Immunosorbent Assay
ESR	Erythrocyte Sedimentation Rate
IQR	Interquartile Range
ISM1	Isthmin-1
NF-κB	Nuclear Factor kappa B
NK	Natural Killer cells
NKT	Natural Killer T cells
RA	Rheumatoid Arthritis
RF	Rheumatoid Factor
ROC	Receiver Operating Characteristic
SD	Standard Deviation
Th17	T helper 17 cells
anti-CCP	Anti-Cyclic Citrullinated Peptide
